# Broadening the Socio-Technical Horizons of Health Informatics

**DOI:** 10.2174/1874431101004010179

**Published:** 2010-09-15

**Authors:** Andrew Georgiou, Sue Whetton

This special socio-technical supplement of the *Open Medical Informatics Journal* showcases a number of innovative and unique research approaches that highlight the current scope of socio-technical perspectives in the health informatics discipline. In just three short decades the discipline of health informatics has evolved from a primary focus on the “application of computers to all fields of medicine – medical care, medical education and medical research” [1]  to an increasing focus on socio-technical issues where “people and organizational issues are critical” (page 79) [2]. The incorporation of socio-technical perspectives arose as researchers sought to analyse the reasons behind the limited successes of many early health information management systems. The application of socio-technical analyses demonstrated that effective systems implementation required an appreciation of both technological and organisational factors. While technical elements continue to be core to health informatics knowledge, socio-technical research seeks to further our understanding of the organisational and cultural factors inherent in the introduction of information management systems into the health care environment. Early socio-technical research included reflections both on its role in knowledge development and its application to the practice of health informatics [3-5]. Research and discussion often took the form of a critique of the established technology focussed approaches. However, as Coiera notes, “for the contribution of socio-technical systems thinking to be more than simply a means of critiquing current practices and systems, it needs to also contribute to the process of developing new and more effective systems” (page S98) [6].

The papers in this supplement highlight how many of today’s researchers have adopted a socio-technical focus as an established and credible methodology, not just for critiquing past mistakes, but for developing an understanding of key success factors in the development and implementation of new systems. The papers also show how researchers are using the basic concepts and multi-method socio-technical approaches, and applying these to a range of research situations. Chhanabhai and Holt demonstrate the universal relevance of socio-technical perspectives in their analysis of information and communications technologies in developing countries [7]. The authors present an overview of current methods through which healthcare information is communicated to the public within these countries. Niazkhani applies socio-technical analyses to a clinical environment. This exploratory research draws on socio-technical theories to provide insights into the differences between the perceptions of surgical and non-surgical clinicians in relation to Computerised Provider Order Entry (CPOE) systems [8]. The papers by Scott [9] and Li [10] highlight the increasing interest of today’s researchers in making connections between academic research and the professional environment. Li’s paper seeks to develop a model that will bring together the theory and practice of socio-technical perspectives in health informatics. Scott also investigates this area as a means of constructing an innovative socio-technical assessment tool for health information system implementations.

The authors contributing to this special edition also highlight the multi-disciplinary, interrelated character of health informatics. The papers by Borycki and Kushniruk [11], and Cummings and Turner [12] demonstrate how a melding of socio-technical perspectives with other approaches can offer new insights for health informatics practitioners. Borycki and Kushniruk combine socio-technical and cognitive approaches to facilitate a more holistic understanding of the impact of health information systems [11], while Cummings and Turner adopt a multi-disciplinary approach in their critical review of current research and evaluation practices [12].

Contributors to this supplement also expand the focus of socio-technical systems research from interaction between clinicians and their systems to explore ways in which other users interact with information management systems. Cummings and Turner note that users of socio-technical systems are no longer restricted to health professionals and highlight the role of patients (or consumers) as important users in their own right [12]. The increasing emphasis on consumer focussed health care creates new challenges for health informatics as consumers take responsibility for managing their own health and well-being supported by information and communication systems.

The contributions to this supplement also point to links between the micro-environment of socio-technical systems and the macro-environment of power, politics and ethics. This is flagged by Chhanabhai and Holt who incorporate concepts of equality and empowerment into their discussion [7], while Borycki and Kushniruk raise the possibility of extending their analysis to incorporate broader social processes and the critical role they play in health information system utilisation, performance and their contribution to patient care [11].

The final paper by Whetton and Georgiou [13] considers the influence of socio-technical perspectives, research and practice within the discipline of health informatics. They note the limited discussion of the philosophy, theory and socio-technical perspectives within health informatics, arguing that instead of a solid theoretical base from which to describe, study and understand human-information technology interactions, we continue to have fragmented, and unelaborated understandings. While socio-technical perspectives within the wider academic research community have begun to explore the interaction between the information system and the broader social environment, the focus in health informatics remains at the level of the immediate environment. In the increasingly complex and interconnected contemporary health care environment, now may be the time to broaden the focus of socio-technical perspectives in health informatics.
